# Compressive Strength, Thermal Conductivity, Vapor Permeability and Specific Heat of Hemp-Lime Composites Varying in Density for Wall, Roof and Floor Applications

**DOI:** 10.3390/ma18214958

**Published:** 2025-10-30

**Authors:** Michał Gołębiewski, Barbara Pietruszka, Wojciech Piątkiewicz, Michał Kubiś, Olena Oleksiienko

**Affiliations:** 1 Faculty of Architecture, Warsaw University of Technology, Koszykowa 55 St., 00-659 Warsaw, Poland; 2 Department of Thermal Physics, Acoustics and Environment, Building Research Institute, Ksawerów 21 St., 02-656 Warsaw, Poland; b.pietruszka@itb.pl; 3 Faculty of Civil Engineering, Warsaw University of Technology, Armii Ludowej 16 Av., 00-637 Warsaw, Poland; wojciech.piatkiewicz.dokt@pw.edu.pl; 4 Institute of Heat Engineering, Faculty of Power and Aeronautical Engineering, Warsaw University of Technology, Nowowiejska 21/25 St., 00-665 Warsaw, Poland; michal.kubis@pw.edu.pl; 5 Ukrcertification LLC, Volodymyr Stelmakh 62D St., 33018 Rivne, Ukraine; mb-elena@ukr.net

**Keywords:** hemp-lime composite, compressive strength, thermal conductivity, water vapor permeability, specific heat, sustainable materials, sustainable construction

## Abstract

Hemp-lime composites, characterized by pro-environmental features, offer an interesting alternative to traditional building materials. The properties of these materials depend on many factors, leading to uncertainty regarding the results obtained when designing building envelopes. Volumetric density—a key factor—depends on the manufacturing technique adopted, among other things, and can be controlled. In this study, materials for use in partitions constituting the building envelope were produced at various densities through varying degrees of compression and subjected to tests to determine their compressive strength, thermal conductivity and water vapor permeability. The values obtained were in the wide ranges of 0.05–1.50 MPa, 0.0671–0.1339 W/(m∙K) and 3.61–10.10, respectively. Furthermore, the specific heat was determined to be 1178–1260 at 60 °C and 773–863 J/(kg∙K) at 30 °C. The test results clearly indicate the dependence of the tested properties on the mixture composition, volumetric density and composite structure. The results of this study demonstrate the suitability of the considered material for construction applications and the significant variations in its properties. Understanding the relationships between the properties of different composites and their degree of compaction can facilitate the design process and help in modeling the mechanical and thermal behaviors of partitions and entire buildings.

## 1. Introduction

### 1.1. Context and Purpose

The protection of natural resources that determine the conditions of life on Earth requires innovations in the field of architecture and construction, considering the significant impact of this sector on the state of the natural environment [1] and the expected intensive environmental transformation processes caused by progressive urbanization [2]. In conventional buildings, as the greatest energy consumption (and, therefore, usually the greatest environmental impact) occurs during the use phase [3], energy efficiency is of key importance. Predominantly used thermal insulation materials (expanded polystyrene, mineral wool) are generally characterized by a relatively high embodied energy and carbon footprint [4]. The growing demand for materials, as well as the need to reduce the consumption of non-renewable resources and harmful technological processes, has led to the search for alternative materials, mainly those of organic and recycled origin [5], with cellulose fibers [6] and straw bales [7] having been commonly used in this context. Nevertheless, some technical aspects limit the wider use of these materials in construction, including issues relating to fire resistance and durability, high costs or the variability of their properties.

This study aims to broaden and update knowledge on the properties of one of the fastest developing (yet still niche) alternative material solutions, namely, hemp-lime composite, commonly referred to as hempcrete. The hemp-lime composite consists of hemp shives and a mineral binder, and requires water for setting. Hemp shives are produced through the decortication and fragmentation of hemp stalks into small particles (usually 0.05–20 mm long). The resulting aggregate has been shown to exhibit a porosity of approximately 76% [8], as well as one of the lowest thermal conductivity coefficients—measuring 0.051 W/(m·K)—among commonly studied plant-based aggregates [9]. Hemp shives are mixed with a mineral grout, which forms the matrix of the composite, primarily providing cohesion, strength, and protection of the plant-based aggregate against biodegradation [10,11]. The most-used binder consists predominantly of hydrated lime, along with hydraulic binders (mainly cement) and pozzolanic binders [12,13,14,15,16,17,18]. Researchers have assessed various new binder compositions, such as those utilizing natural hydraulic lime [13], prompt natural cement [14], metakaolin [15] and magnesium phosphate cement [19]. Due to its good thermal insulation [20] and acoustic properties [21], significant transpirability and hygroscopicity [22], and low carbon footprint [23], hempcrete has gained popularity as an alternative building material [24]. The most widespread application method involves placing the material in a two-sided formwork followed by compaction through manual or mechanical tamping; however, it may also be sprayed onto one-sided formwork or prefabricated in the form of blocks (for bricklaying) or panels (for assembly) [25,26,27]. Although the most common application of hemp-lime composite is as a filling for wooden frame structures in the external walls of buildings, it demonstrates a broad range of applications, including the thermal retrofitting of existing external partitions, the filling of internal partition walls, as well as thermal insulation of floors and ceilings [28]. Since there are no uniform guidelines for the production of or building with hemp-lime composites, contractors must rely on instructions provided by component manufacturers, practical construction manuals and the experience of specialized workers.

The relationships between volumetric density and compressive strength, thermal conductivity and water vapor permeability are key to the architectonic design process, as the density of hempcrete may vary to great extent with the proportions of the ingredients or compaction level. Such knowledge, in the absence of standards on building with hempcrete, has practical significance, as it allows designers to check whether specific national legal requirements for building partitions or entire buildings are met (e.g., thermal protection, energy efficiency, or condensation risk), as well as facilitating design optimization.

### 1.2. Factors Influencing Compressive Strength

Compressive strength is one of the primary characteristics of hemp-lime composites that has been investigated in the existing literature [16,17,18]. It can vary widely, ranging from 0.06 MPa [19] to as high as 4.74 MPa [29]; however, it is most reported to be in the range of 0.1–1.2 MPa [19,25,30,31,32,33]. The primary factor influencing compressive strength is the material’s volumetric density, which depends on the proportion of constituents, among other things [13,31,32,34]. In [35], the authors demonstrated that doubling the binder-to-hemp mass ratio resulted in an approximately 50% increase in density and more than a twofold increase in compressive strength. Similar findings were reported in [19]. Another significant variable affecting density—and, therefore, compressive strength—is the degree of material compaction [26]. Due to the relatively low density of hempcrete and the irregular, angular shape of its aggregate, the material does not self-compact under its own weight and requires manual or mechanical densification [36]. A study [37] demonstrated that a 19% increase in density (due to compression) led to a 67% increase in compressive strength. Similar observations were presented in [38,39]. Compressive strength can also be modified through the appropriate selection of a binder; for example, it has been shown that using prompt natural cement with the addition of citric acid led to an increase in strength by up to 40%, in comparison with commercial preformulated binder, while maintaining the same density [31]. In [40], it was reported that characteristics of the aggregate also influence the compressive strength of the composite; in particular, a predominance of short particles promoted greater strength.

### 1.3. Factors Influencing Thermal Conductivity

The thermal conductivity coefficient values determined in numerous studies range from 0.06 to 0.16 W/(m·K) [19,29,30]. An analysis of the literature indicated that the thermal conductivity of hempcrete is predominantly influenced by its volumetric density [13,28,30,31,32]. As mentioned above, the density of the material is primarily determined by the mass ratio of the components and the degree of compaction [29,33,34]. A reduction in the binder-to-hemp ratio from 2.5:1 to 1.5:1 was observed to decrease the thermal conductivity coefficient by 25% [33]. Similar results were obtained in [35], where a change from 2.6:1 to 1.8:1 resulted in a 30% reduction in the thermal conductivity coefficient. In studies involving manual material placement [36], an increase in compaction level (reduction in volume compared with the uncompacted state) from 30% to 60% led to an increase in density by approximately 18% and an 8% increase in thermal conductivity. In [29], significant compression was achieved through the use of a device generating stresses in the range of 0.6–1.0 MPa, resulting in a threefold reduction in volume and a 35% increase in the thermal conductivity coefficient. Studies on the impact of including mineral additives in the binder composition on thermal conductivity of hempcrete have yielded inconclusive results. Some publications suggest a marginal influence of mineral additives [30,32], while in other studies, the use of binders based on starch or magnesium compounds significantly improved the thermal insulation properties of the materials [19,37]. The effect of aggregate size on the thermal conductivity of the composite is also ambiguous. In [41], no significant effect of hemp shiv particle size on thermal conductivity was observed; meanwhile, in [40], the use of a smaller aggregate resulted in higher conductivity due to better material compaction. Similarly to other porous thermal insulation materials, increased humidity and temperature has been shown to lead to an increase in the thermal conductivity of hempcrete [12,34,39,42]. The considered composite is characterized by an anisotropic structure. In [36], the thermal conductivity coefficient values were lower when the direction of heat flow was parallel to the direction of material compaction, with the difference reaching up to 16%.

### 1.4. Factors Influencing Water Vapor Permeability

The diffusion resistance coefficient of hempcrete in a RH 50% falls within the 3.6–7.7 range, with values around 5 being the most common in the literature [30,31,43]. In [31], it was shown that the water vapor permeability of hempcrete depends on both the characteristics of its components and their proportions; however, in [30], it was revealed that the micropores of the binder have a small impact, while the characteristics of macropores between hemp shiv particles act as a dominating factor, mainly resulting from the proportions of ingredients and the degree of mixture compaction. In [43], it was found that higher relative humidity induces higher vapor permeability rates of the composite. A similar conclusion was drawn in [40], where the diffusion resistance coefficient of hempcrete was determined for two humidity ranges—namely, 0–50% and 50–94%—and for thick (average length 11.78 mm; average width 4.55 mm) and fine (2.74; 1.42 mm) hemp shiv particles. The results were in the ranges of 12.18–12.37 (lower RH) and 5.57–4.94 (higher RH), respectively, highlighting higher permeability at higher RH and the relatively minor effect of hemp shiv particle size on the tested coefficient.

### 1.5. Factors Influencing Specific Heat

The specific heat of hemp shives (at 23 °C) was determined to be approx. 1250 J/(kg·K) [39]. The relevant literature has reported significantly diverse specific heat values for hemp-lime composites, ranging from 300 to 1601 J/(kg·K) [39,40,44]. The factors causing such discrepancies seem to be not only related to the compositions of the composites, but also the research methodology. According to [39], indirect methods based on the results of thermal conductivity and density tests may underestimate the values obtained, when compared with direct measurements. The authors of [44] suggested that the moisture content of the material may lead to atypical results. In [39], a hemp-lime composite tested using two direct methods showed specific heat values of 905 and 911 J/(kg·K) (at 20 °C), whereas the indirect method yielded a result that was 18% lower. Measurements using indirect methods also demonstrate sensitivity to the material’s anisotropy: for a hemp-lime composite with a density of approximately 400 kg/m^3^, specific heat values of 620 and 840 J/(kg·K) were obtained for heat flow parallel and perpendicular to the dominant orientation of hemp shiv particles, respectively [42]. On the other hand, some tests carried out using the indirect method resulted in relatively high values of specific heat: in [45], for wet (RH 50%) and dry composites with a 1:1 binder-to-hemp mass ratio, the results ranged from 1398 to 1557 J/(kg·K) and from 1250 to 1421 J/(kg·K), respectively. The type of binder and the shiv particle size seem to be of lesser importance. According to [30], the use of hydraulic binders resulted in only slightly higher specific heat when compared with binders based on hydrated lime with pozzolanic additives. Comparison of specific heat values measured using a direct method for two different gradations of hemp shives revealed values of 1575 and 1601 J/(kg·K) for fine and coarse aggregates, respectively, indicating a marginal influence of hemp shiv particle size on the tested feature [40]. To increase the specific heat of the composite—and thus, the thermal capacity of building partitions—the effect of PCM addition has also been investigated [46].

## 2. Materials and Methods

### 2.1. Mixtures and Samples Preparation

Hemp-lime composite samples were produced from hemp shiv and a preformulated binder (formulated lime of class FL A 3.5 compliant with EN 459-1:2015 [47]) obtained from European producers (Saint-Gobain, Paris, France), the technical sheets for which are available at [48,49], accordingly. Only the general binder’s composition is given: air lime (98% Ca(OH)_2_), 75%; hydraulic binder, 25%; and minor additives [50]. Water at a temperature of 20 ± 2 °C was obtained from the Warsaw water supply network.

**Figure 1:**
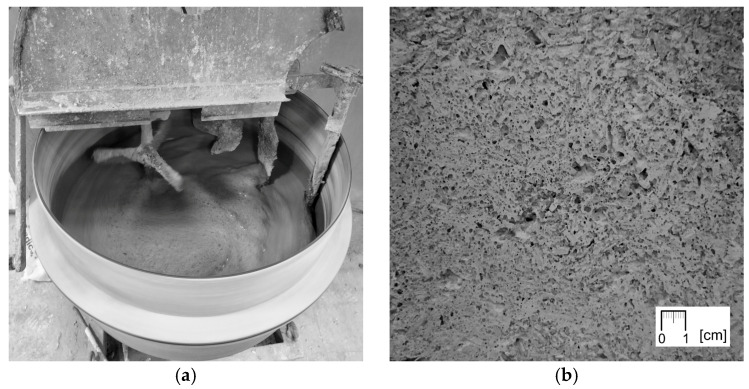
(**a**) Foaming of the F mixture grout during mixing; (**b**) structure of F690 sample with visible characteristic pores in the matrix resulting from the release of air from the foamed mixture.

The method of sample preparation was adopted to reproduce the procedure and conditions prevailing on construction sites of hempcrete buildings erected by tamping—the dominant technique in small, individual projects [28]. Three series of samples were produced from mixtures with varying proportions of constituents, corresponding to three main building applications: R—horizontal insulation of roof (above the structural ceiling/between the rafters) or raised floor (between the beams), W—wall infill (for timber/steel frame) and F—floor on ground (material which transfers loads into the ground). The proportions of components were adopted from the instructions of a European manufacturer (Table 1) [50]. The ingredients were mixed in a mixer with a vertical axis of rotation. First, water and binder were mixed for 3 min, following which hemp shiv was added and mixed for another 5 min. During mixing of grout F, intense foaming occurred (Figure 1a), which was not observed on such a scale during the preparation of other mixtures. As a result, the F samples presented a specific porous structure (Figure 1b).

Mixtures of a certain mass were placed in molds of a certain volume and perchance compressed by tamping. In the case of series R and F (material applied in the form of a horizontal layer), the samples with the lowest density were obtained by completely filling the volume of the molds, without compaction. In the case of the W series (material applied in the form of a vertical layer) the procedure was similar, but slight compression was applied to eliminate the resulting voids. The lowest adopted volumetric density was the first one ensuring that the sample would not spontaneously fall apart during demolding. Then, subsequent samples of all series were made by placing more mixture in the molds (with the volumetric density increased by 0.3 kg/m^3^), which required increasingly greater compaction. Ultimately, the samples with highest density were obtained, i.e., those that could not be further compacted manually. Due to the foaming of mixture F, subsequent samples of this series were produced at approximately 15-min intervals, which allowed for the release of more air and obtaining higher volumetric densities (otherwise, compressing the mixture to the intended volume would not have been possible).

For the W series, 10 samples were produced while, for the R and F series, 9 samples were produced for each tested feature (except for specific heat). The samples were demolded after 1–2 days and seasoned for 90 days: the first 7 days at 23 °C and RH 80% (except for the samples for compressive strength tests, which were kept in such conditions for 28 days), and the remaining period at 23 °C and RH 50%. For the specific heat tests, 5 cubes of 0.05 × 0.05 × 0.05 m for each series were cut from the 0.3 × 0.3 × 0.08 m blocks of the lowest density (from different, distant places) and conditioned as described above. Prior to testing, these samples were pulverized and dried (as described in Section 2.5).

### 2.2. Compressive Strength

**Figure 2:**
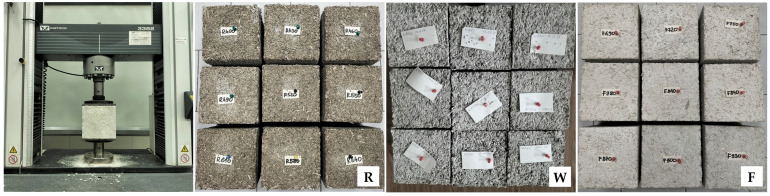
Instron 3382 machine and samples of all series prepared for compressive strength tests.

The compressive strength of the material was determined through a destructive compression test carried out on 28 cubic samples with dimensions of 0.15 × 0.15 × 0.15 m, at 90 days after their production, using an Instron 3382 testing machine (Instron, Norwood, MA, USA) with a maximum load capacity of 100 kN (Figure 2). The samples were uniformly compressed at a jaw displacement rate of 5 mm/min, in the direction corresponding to the loads to which the material would be subjected to in the intended structural application. To ensure uniform force distribution, an 8 mm thick steel plate was placed on each sample and in contact with the moving press head. Parameters such as force, displacement, and stress were recorded using the Instron Bluehill 2 (version 2.14) software, which generated real-time stress–strain curves. Prior to testing, a preload of approximately 0.02 kN was applied to minimize the impact of surface irregularities, after which the displacement measurement was reset to zero. The mass of the steel plate was excluded from calculations, as it accounted for less than 2% of the lowest recorded compressive strength value.

There is no standard or generally accepted method for determining the compressive strength of hemp-lime composites. In this study, different methods were used depending on the mechanical behavior of the material being tested. Only the F series samples showed a characteristic point of maximum stress (followed by its reduction), which was thus used to determine ultimate compressive strength. As such a point did not occur in the stress–strain curves of the W series samples, the strength of this series was determined according to the method proposed in [35], i.e., at the point where the instantaneous stiffness falls to 25% of the recorded maximum value based on a 20-point moving average. The distinct deformation characteristics of R series samples required yet another analytical approach. For this series, the compressive strength was determined at the point where the samples reached 5% deformation, which was used to establish the serviceability limit state in [51]. Additionally, to compare the results of samples from all series, the strength based on the stress recorded at 5% strain was also presented (in relation to volumetric density) for the series F and W samples.

### 2.3. Thermal Conductivity

**Figure 3:**
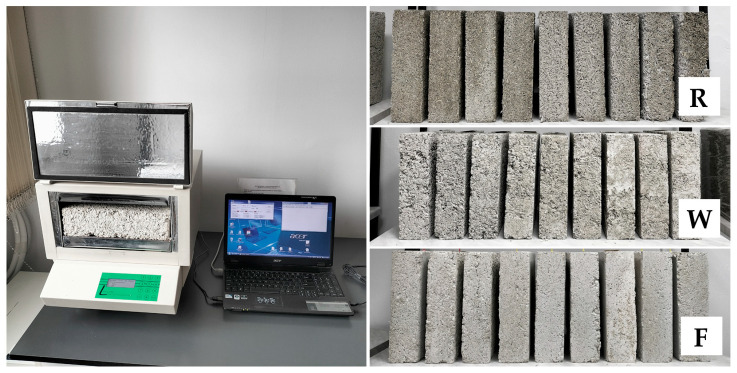
Fox 314 apparatus and samples of all series prepared for thermal conductivity tests.

The thermal conductivity tests were carried out on 28 rectangular samples with dimensions of 0.3 × 0.3 × 0.08 m, at 90 days after their production, using an FOX 314 plate apparatus (TA Instruments, New Castle, DE, USA) with heat flux density sensors (Figure 3), in accordance with the EN 12664:2002 [52]. The mass of the samples was stabilized prior to testing. The temperature in the test chamber was maintained between 22.2 and 22.7 °C. The measurements were made at an average sample temperature of 10 °C, a temperature difference across the sample thickness of 20 K, heat movement from the bottom to the top (perpendicular to the direction of sample compaction, i.e., in the direction of heat flow in the building partition). The change in the mass of the samples during the test did not exceed 0.2%.

### 2.4. Water Vapor Permeability

**Figure 4:**
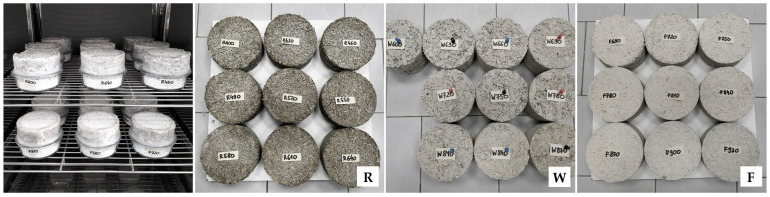
Sealed samples in climatic chamber and samples of all series prepared for water vapor permeability tests.

The water vapor permeability tests were carried out on 28 cylindrical samples with a diameter of 0.128 m and a height of 0.05 m (R and F series) or 0.08 m (W series), at 90 days after their production, using the “Cup” method in accordance with the EN ISO 12572:2016 [53], with minor modification: instead of steel vessels, samples were placed onto glass vessels (Figure 4). The W series samples were cut from the 0.3 × 0.3 × 0.08 m blocks to ensure correct direction of vapor flow, i.e., perpendicular to the direction of sample compaction. The mass of the samples was stabilized prior to testing. Samples were placed onto vessels filled with calcium chloride and sealed with liquid wax, leaving a surface with 0.1 m diameter at the top. The vessels were weighed and placed in a chamber (Constant Climate Chamber HPP Cooled Incubator IPP^PLUS^ by Memmert, Schwabach, Germany) at temp. 23 ± 1 °C and RH 50 ± 5%. Vessels were weighed at intervals of 1–3 days until the calculated change in mass per hour for each individual vessel in each of the previous 5 measurements was within 5% of the mean change in mass of the vessel from these measurements. In the case of W series samples, due to problems with mass stabilization of 3 samples, an average from 4 measurements was used in calculations instead of 5 (advised by the standard); it is believed that the impact of this change was insignificant for the results. After 15, 17 and 16 days, the tests of the R, W and F series samples had completed. The coefficient of water vapor permeability was calculated according to the equations in the EN ISO 12572:2016 standard [53]. The coefficient of diffusion resistance was determined as the ratio of the vapor permeability of air (δ_a_ = 720 × 10^−6^ g/(m·h·Pa)) to the vapor permeability of the material.

### 2.5. Specific Heat

**Figure 5:**
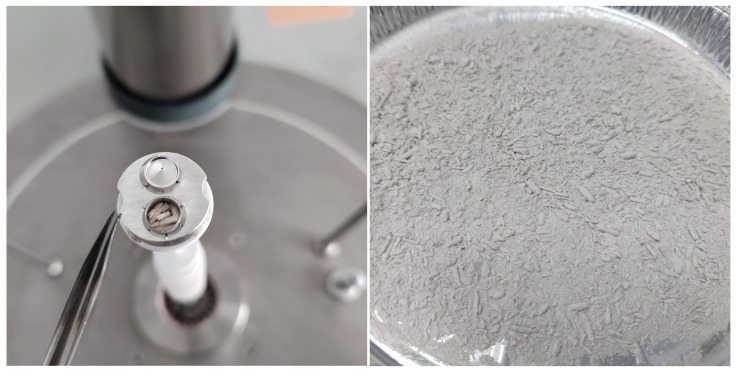
Hemp shiv sample in DSC crucible and example crushed sample of hemp-lime composite prepared for specific heat testing.

Samples for testing with a differential scanning calorimeter (DSC) required several stages of preparation, consisting of grinding, drying, collecting, and placing in a crucible. Pre-prepared 0.05 × 0.05 × 0.05 m cubes were split into many smaller pieces. Then, about three-quarters of each original sample was crushed using a high-speed impact mill (IKA A11) (IKA-Werke GmbH & Co. KG, Staufen, Germany). The samples were ground in two stages. The total grinding time was set at 45 s, with the first stage lasting 30 s and the second stage lasting 15 s. Between the first and second stages, the grinding state of the sample was checked. There were no noticeable differences between all samples. Each sample obtained in this way was spread evenly on an aluminum tray with a diameter of approx. 20 cm, forming a thin layer. An example sample after grinding is shown in Figure 5. The trays were placed in a dryer at 70 °C for 72 h. After this time, a portion of powder of each sample was taken and poured into a DSC crucible of approx. 5 mm diameter (Figure 5). Rare, unusually long particles were removed to avoid disturbing the sample structure during pressing. The pressed samples occupied about half the volume of the DSC crucible, weighed approx. 16–18 mg and were considered representative. For each type of mixture, 5 samples were prepared. Additionally, tests were carried out on hemp shives alone, which were pre-crushed and dried under similar conditions as the composite samples. Three hemp shiv samples were prepared by loosely placing several particles in a Pt-Rh crucible, resulting in a sample mass of approx. 6.5 mg. To ensure that the samples remained dry, the temperature program was set to consist of repeated heating and cooling cycles.

The specific heat of hemp-lime composites was determined using a DSC 404 F1 Pegasus (Netzsch) apparatus (NETZSCH-Gerätebau GmbH, Selb, Germany) (Figure 5). For this purpose, Pt-Rh crucibles and sapphire as a reference material were used, and an inert gas atmosphere was provided using argon with a flow rate of 20 mL/min. The test was carried out in the temperature range from 30 to 70 °C in three heating–cooling cycles, where the heating rate was 2 K/min and the cooling rate was 2 K/min. An isotherm of t = 10 min was applied between the cooling and heating stages, which allowed a steady state to be reached before the next heating cycle. The data for determining the specific heat was obtained from the last heating cycle. The samples were weighed with an accuracy of 0.0001 mg (Supermicro, Sartorius AG, Göttingen, Germany). At the end of the measurement series, a reference sample in the form of amorphous sapphire was tested, which was treated as a sample with unknown specific heat to verify the measurement accuracy. In the tested range, the largest difference recorded was less than 1.5% in the upper temperature range while, in the lower temperature range, this difference was less than 1%.

## 3. Results and Discussion

### 3.1. Compressive Strength

**Figure 6:**
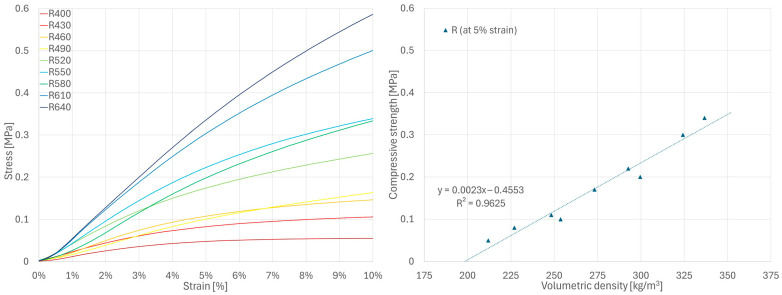
Results of compressive strength tests for R series samples.

**Figure 7:**
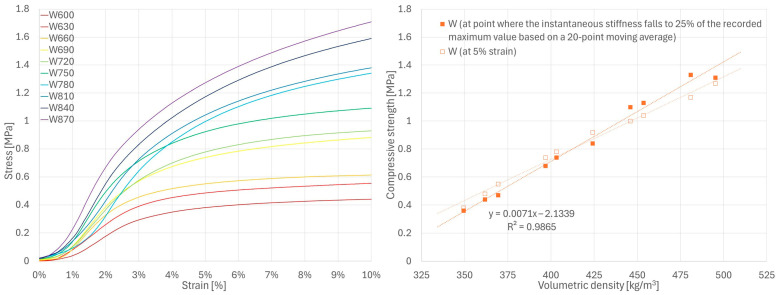
Results of compressive strength tests for W series samples.

**Figure 8:**
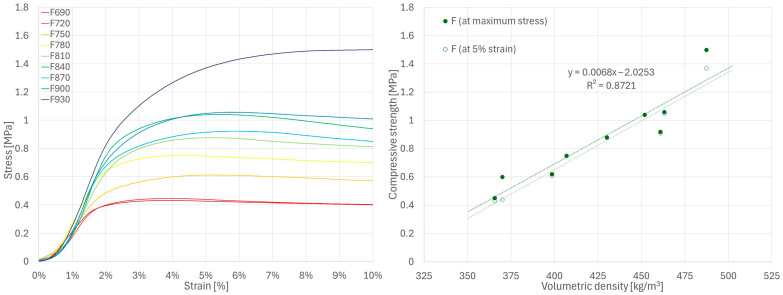
Results of compressive strength tests for F series samples.

Despite using the same test procedure for all mixtures, their stress–strain curves show clear differences. The R series specimens showed significant deformations at low stress levels (Figure 6), while the W series specimens showed a steeper stress–strain curve with noticeable quasi-linear regions (Figure 7). In both series, the stress increased continuously until the test was terminated at 10% strain (due to visible material damage). The F series curves showed significantly increased stress levels in the initial loading period and reached a characteristic maximum, after which the stress was reduced with a further increase in strain (Figure 8). The higher stiffness of the F series samples indicates a greater contribution of the binder to the transfer of initial loads.

The compressive strength values determined using different methods for series R, W and F samples are shown in Figure 6, Figure 7 and Figure 8, respectively. Detailed values for all samples are presented in Appendix A.1. For all tested series, a close-to-linear relationship was observed, indicating an increase in strength with increasing volumetric density and, consequently, a more compact composite structure.

These results are generally consistent with the values presented in the literature for various types of hemp-lime composites [19,25,30,31,32,33]. As expected, the R series samples, which were characterized by the lowest density, showed the lowest strength; this is related to the lowest binder content in the mixture and, consequently, small contact surfaces between aggregate particles. The W series samples showed slightly higher strength than the F series samples, despite the lower binder-to-hemp mass ratio in the mixture. Analysis of the F series stress–strain curves indicates that the ultimate strength—determined using the same method as for W (i.e., the rate at which the 25% maximum instantaneous stiffness criterion is achieved [35])—would occur at 2–3% strain, while in the W series this point was shifted toward higher strains with increasing sample density, reaching values in the range of 4–7%. In the case of the F series samples, this criterion was met more rapidly, and the stress value at this point remained lower than the maximum stress recorded during testing. For this reason, the method adopted for the W series does not seem to be the most appropriate for the F series. However, even when using the criteria of highest recorded stress or stress at 5% strain (Figure 8), the results for the F series specimens were still lower than those of the W series. It should therefore be assumed that composite W, despite its lower binder-to-hemp ratio, is characterized by higher strength than composite F due to differences in structure, or that the method of determining the strength for the W series samples overestimated their results.

**Figure 9:**
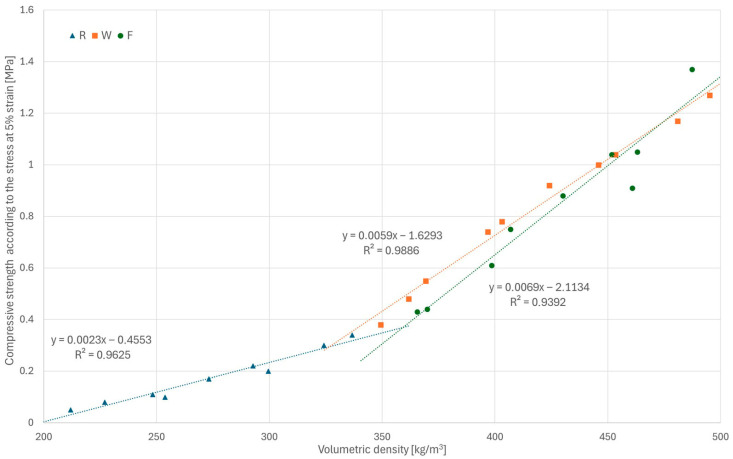
Stress values at 5% displacement in relation to density for all samples.

To directly compare the results for all series, the strength calculated based on the stress values at 5% strain in relation to the volumetric density of the samples is shown in Figure 9. It is worth noting that this method yields values similar to those obtained using methods selected specifically for individual series (W and F). According to the obtained relationships, an increase in composite density leads to a more significant increase in strength in composites with a higher binder-to-hemp mass ratio.

**Figure 10:**
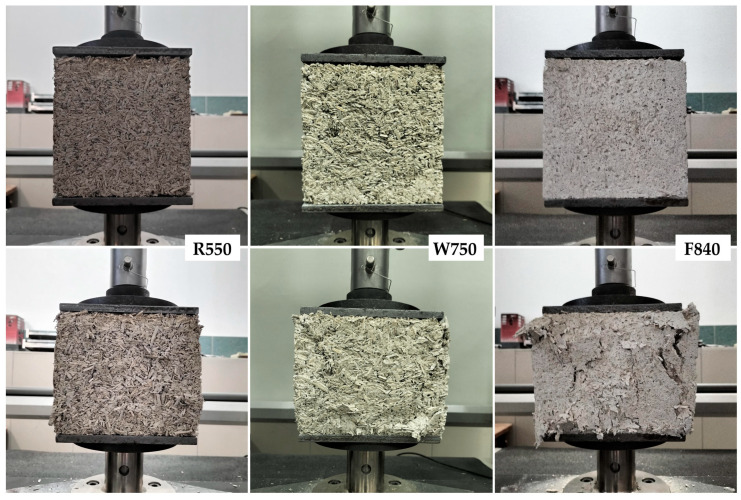
Example average density samples from each series before and after destructive testing.

Example samples from each series before and after testing, showing the nature of their failure, are presented in Figure 10.

### 3.2. Thermal Conductivity

**Figure 11:**
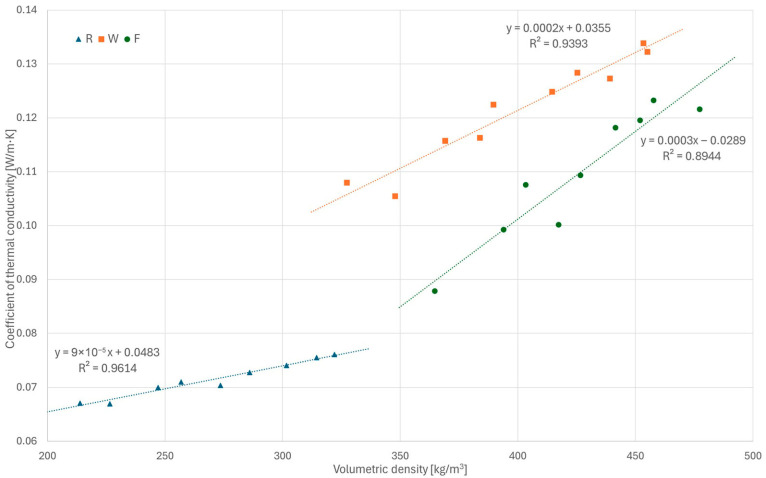
Results of thermal conductivity tests for all series.

The results of the thermal conductivity tests are presented in Figure 11, while detailed values are given in Appendix A.2. In all tested series, a clear relationship was observed, indicating an increase in conductivity with increasing volumetric density; similar results have been reported in [13,28,30,31,32]. The increase in conductivity becomes steeper with increasing binder-to-hemp ratio.

**Figure 12:**
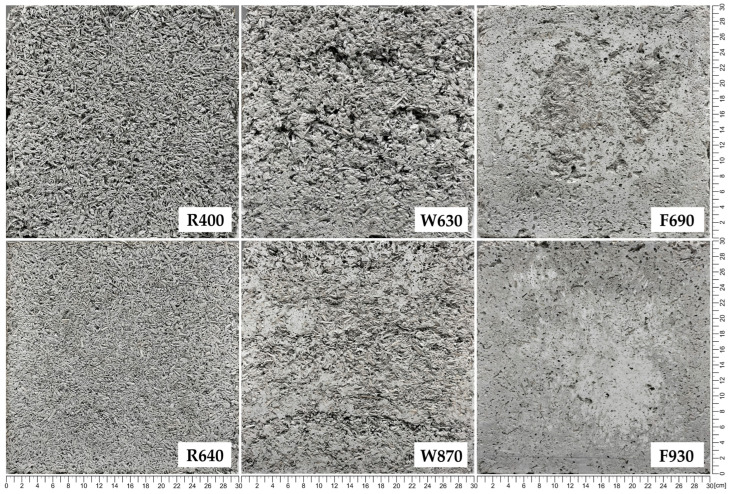
Surface photos of the lowest and highest density samples for all series, showing differences in their composite structures.

The R series samples showed the lowest thermal conductivity (0.0671–0.0761 W/(m∙K)) due to highest share of hemp shiv and, consequently, the lowest density. Increasing the binder share in the W and F series (compared with the R series) resulted in increased thermal conductivity (similarly to [33,35]); however, unexpectedly, the W series samples showed higher thermal conductivity than the F series samples at similar densities—0.1080–0.1339 and 0.0879–0.1233 W/(m∙K), respectively—despite the lower binder-to-hemp ratio in the W mix. This may be due to different pore characteristics of these composites (foaming of the F mixture, as described in Section 2.1). Visual inspection (Figure 12) shows that the macropores of the W samples (especially the low-density ones) are larger and interconnected, forming continuous voids, which can potentially enhance heat transfer by convection; in contrast, in the F samples, the macropores are more evenly distributed, smaller and do not form continuous voids. The observed trends indicate that the difference between W and F values decreases with increasing compactness. However, further investigation of this phenomenon is required to clarify its cause. The obtained thermal conductivity values of the composites are generally in line with literature data [19,29,30]. In a general comparison with bio-based thermal insulation materials [5,6,7], hempcrete showed slightly higher thermal conductivity coefficients in most cases due to its mineral matrix. This requires thicker material layers to provide similar thermal resistance, although higher volumetric density is expected to provide higher thermal capacity of partitions, which is also important regarding the thermal protection of buildings.

### 3.3. Water Vapor Permeability

**Figure 13:**
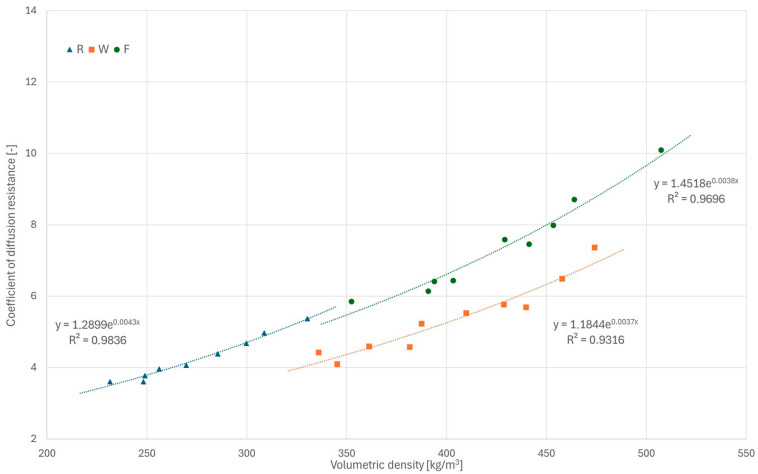
Results of water vapor permeability tests for all series.

The results of the water vapor permeability tests are presented in Figure 13, while detailed values for all samples are given in Appendix A.3. For all tested series, an increase in the diffusion resistance coefficient was observed with increasing volumetric density, and the relationship was close to exponential in the tested density ranges. The R series samples were characterized by the lowest diffusion resistance coefficients (3.61–5.37) and F series samples by the highest (5.87–10.10). These results generally fall within the range of values previously reported in the literature [30,31,40,43]. The combined results for the R and F series form a well-correlated exponential trend (R^2^ = 0.9896) despite significantly different component ratios, which may suggest the volumetric density as a main parameter for estimation of the diffusion resistance coefficient. On the other hand, the results for the W series samples (4.10–7.37) are clearly lower than those for F and do not fall within the “R + F trend,” which may be the result of a more heterogeneous structure of this type of composite. Due to compaction method and the consistency of the mix itself, the voids in W composite samples tended to have a more random shape and size (forming continuous channels, allowing for easier vapor flow), while the macropores of F composite samples were less differentiated and more evenly distributed (Figure 13). These observed characteristics demonstrate the importance of macropores between shiv particles for the water vapor transport process (as previously noted in [30]).

### 3.4. Specific Heat

**Figure 14:**
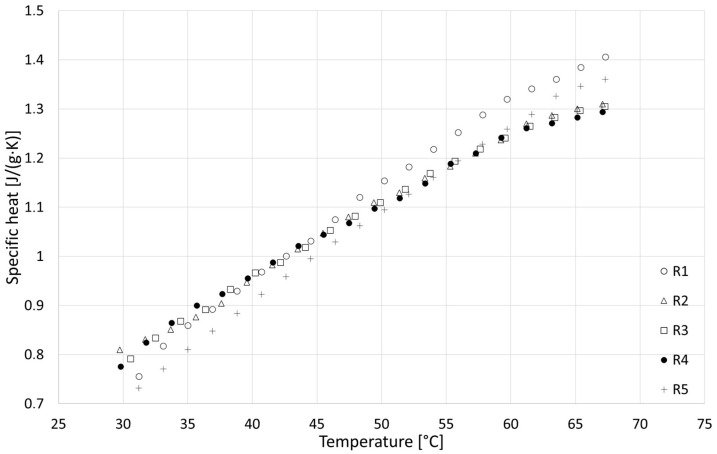
Results of specific heat tests for R series samples.

**Figure 15:**
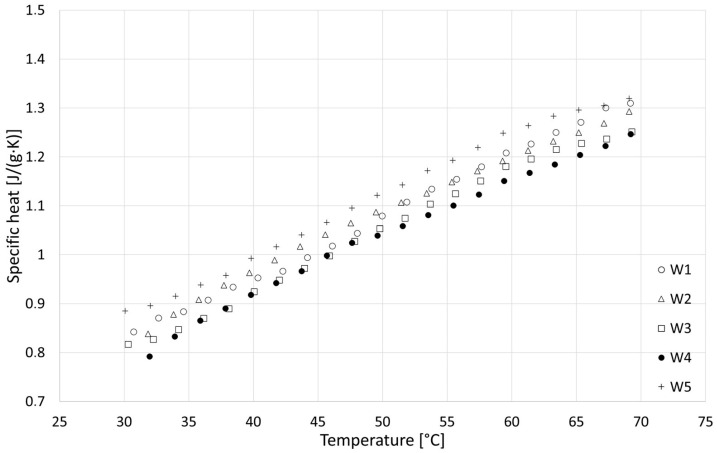
Results of specific heat tests for W series samples.

**Figure 16:**
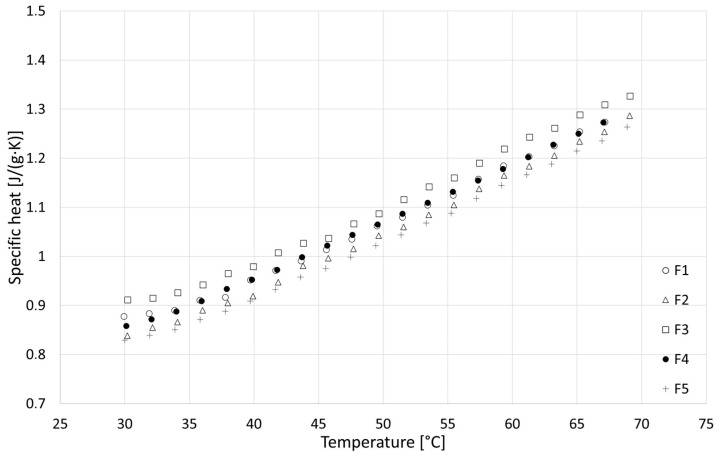
Results of specific heat tests for F series samples.

The results of the specific heat tests are presented in Figure 14, Figure 15 and Figure 16, while detailed values for all samples are given in Appendix A.4. Based on the analysis over three heating cycles, no significant differences were observed between DSC signals from the second and third heating cycles. Throughout the entire measurement range, these differences were within the measurement error range of ±1%. Slightly larger differences were observed between the first and the second heating cycle, which could indicate the removal of residual moisture. On this basis, it can be concluded that determining the specific heat based on data from the last heating cycle is justified and safe. All samples showed similar properties, with specific heat increasing with temperature, and the obtained specific heat values for different types of samples were similar. In the upper temperature range, the R series samples achieved the highest values, while those of the F series samples were the lowest values. In the lower temperature range, the F series samples achieved the highest values and R the lowest. Linear extrapolation to 20 °C revealed mean values for R, W and F series samples of 0.629, 0.710 and 0.729 J/(g∙K), respectively. The obtained results are in the lower range of values found in the literature, close to [42] (indirect method), slightly lower than those in [39] (direct method) and much lower than those in [40] (direct method). These specific heat values, which are generally lower than those reported for various insulation materials of biological origin [3,5], may result in lower-than-expected thermal capacity and higher thermal diffusivity of building partitions made of hempcrete, in effect lowering the thermal inertia of building envelopes. As thermal capacity is considered one of the possible advantages of hemp-lime composites compared with typical fiber insulating materials used in lightweight frame structures, this issue requires further research to gain insights.

**Figure 17:**
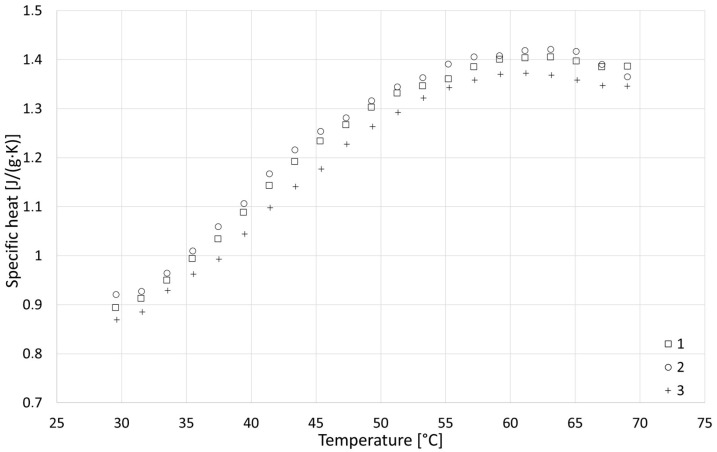
Results of specific heat tests for hemp shiv.

The obtained results prompted an investigation of the specific heat of the plant component itself, the results of which are presented in Figure 17. The observed low specific heat of the aggregate explains the relatively low values obtained for the entire composite, indicating that this factor may be one of the reasons for the large variation in specific heat values of hempcrete reported in the literature [39,40,44]. The relationship between specific heat and temperature was slightly different for hemp shiv samples; while close to linear at lower temperatures, the values started to decrease at T = 55 °C. This may indicate the beginning of a certain transformation or exothermic reaction. Unfortunately, the entire effect was not captured due to the narrow measurement range. Similar characteristics in the upper measurement range can be observed in the case of R samples (with the highest share of hemp shiv).

## 4. Conclusions

The results of the tests performed in this study indicate wide ranges of compressive strength, thermal conductivity and water vapor permeability values of the developed hemp–lime composites, which can be controlled according to the adopted degree of densification, making this material useful in various construction applications. The established relationships between the proportions of components, volumetric density and the main properties of hempcrete may prove useful in building design optimization processes. The data obtained will be used in further research focused on the properties of building partitions and entire buildings constructed using hempcrete technology.

Regardless of the volumetric density, the macropore structure was found to clearly influence the main properties of the tested materials, especially those with a lower degree of densification, leading to uncertainty when aiming to inferring material properties from volumetric density.

Due to the randomness of the plant component characteristics and the adopted production method, hempcrete cannot be considered homogeneous; that is, the concentrations of components and the macropore structure are not strictly controllable, and likely were not entirely evenly distributed throughout the volume of the tested samples. This specificity of the material may explain the results of single samples deviating from the overall trends.

Relatively low specific heat values were recorded for the tested composites, which was mainly due to the low specific heat of the hemp shiv used. It seems advisable to conduct broader research on plant ingredients in this context, considering the influence of raw material production technology on the thermal properties of the resulting materials.

It seems reasonable to establish a universal method for determining the compressive strength of hemp-lime composites, considering their use and static work in the building, which would allow for better comparison of results.

## Figures and Tables

**Table 1:** Characterization of mixes.

Mix Symbol	Hemp/Binder/Water Ratio by Mass	Lowest Initial Volumetric Density (kg/m^3^)	Highest Initial Volumetric Density (kg/m^3^)
R (roof)	1:1:2.15	400	640
W (wall)	1:1.8:3	600	870
F (floor)	1:2.25:4	690	930

## Data Availability

The original contributions presented in this study are included in the article. Further inquiries can be directed to the corresponding author.

## Appendix A

### Appendix A.1

**Table A1:** Detailed results of compressive strength tests of R samples.

Measured Parameter (Unit)	R400	R430	R460	R490	R520	R550	R580	R610	R640
Volumetric density at RH 50% (kg/m^3^)	211.9	227.0	248.3	253.7	273.2	292.7	299.6	324.2	336.7
Volumetric density of dry sample (kg/m^3^)	197.1	211.0	231.9	236.0	253.7	271.3	277.9	300.5	312.1
Compressive strength ^1^ (MPa)	0.05	0.08	0.11	0.10	0.17	0.22	0.20	0.30	0.34

^1^ Determined as the point where 5% strain occurs.

**Table A2:** Detailed results of compressive strength tests of W samples.

Measured Parameter (Unit)	W600	W630	W660	W690	W720	W750	W780	W810	W840	W870
Volumetric density at RH 50% (kg/m^3^)	349.4	361.7	369.3	396.8	403.1	424.1	445.9	453.5	480.9	495.2
Volumetric density of dry sample (kg/m^3^)	318.6	330.2	335.9	361.3	366.3	384.7	404.0	411.0	434.2	447.2
Compressive strength ^1^ (MPa)	0.36	0.44	0.47	0.68	0.74	0.84	1.10	1.13	1.33	1.31
Compressive strength ^2^ (MPa)	0.38	0.48	0.55	0.74	0.78	0.92	1.00	1.04	1.17	1.27

^1^ Determined as the point where the instantaneous stiffness falls to 25% of the recorded maximum value based on a 20-point moving average. ^2^ Determined as the point where 5% strain occurs.

**Table A3:** Detailed results of compressive strength tests of F samples.

Measured Parameter (Unit)	F690	F720	F750	F780	F810	F840	F870	F900	F930
Volumetric density at RH 50% (kg/m^3^)	365.6	370.0	398.5	406.9	430.1	451.8	460.9	463.1	487.3
Volumetric density of dry sample (kg/m^3^)	333.6	338.8	362.9	369.8	390.0	409.1	417.1	420.0	441.9
Compressive strength ^1^ (MPa)	0.45	0.60	0.62	0.75	0.88	1.04	0.92	1.06	1.50
Compressive strength ^2^ (MPa)	0.43	0.44	0.61	0.75	0.88	1.04	0.91	1.05	1.37

^1^ Determined as the point where maximum stress is reached. ^2^ Determined as the point where 5% strain occurs.

### Appendix A.2

**Table A4:** Detailed results of thermal conductivity tests of R samples.

Measured Parameter (Unit)	R400	R430	R460	R490	R520	R550	R580	R610	R640
Volumetric density at RH 50% (kg/m^3^)	213.8	226.6	247.0	256.8	273.6	286.0	301.6	314.4	322.0
Coefficient of thermal conductivity (W/(m·K))	0.0671	0.0670	0.0700	0.0710	0.0704	0.0728	0.0741	0.0755	0.0761

**Table A5:** Detailed results of thermal conductivity tests of W samples.

Measured Parameter (Unit)	W600	W630	W660	W690	W720	W750	W780	W810	W840	W870
Volumetric density at RH 50% (kg/m^3^)	327.3	347.8	369.2	383.9	389.6	414.6	425.2	439.1	455.1	453.3
Coeff. of thermal conductivity (W/(m·K))	0.1080	0.1055	0.1157	0.1163	0.1225	0.1249	0.1284	0.1273	0.1323	0.1339

**Table A6:** Detailed results of thermal conductivity tests of F samples.

Measured Parameter (Unit)	F690	F720	F750	F780	F810	F840	F870	F900	F930
Volumetric density at RH 50% (kg/m^3^)	364.6	393.9	417.3	403.3	426.6	441.5	451.9	457.6	477.3
Coefficient of thermal conductivity (W/(m·K))	0.0879	0.0993	0.1002	0.1076	0.1094	0.1182	0.1196	0.1233	0.1216

### Appendix A.3

**Table A7:** Detailed results of water vapor permeability tests of R samples.

Measured Parameter (Unit)	R400	R430	R460	R490	R520	R550	R580	R610	R640
Volumetric density at RH 50% (kg/m^3^)	231.6	249.0	248.2	256.2	269.7	285.5	299.8	308.8	330.3
Coefficient of diffusion resistance (-)	3.61	3.78	3.61	3.96	4.07	4.38	4.68	4.97	5.37

**Table A8:** Detailed results of water vapor permeability tests of W samples.

Measured Parameter (Unit)	W600	W630	W660	W690	W720	W750	W780	W810	W840	W870
Volumetric density at RH 50% (kg/m^3^)	335.9	345.2	381.5	361.1	387.4	409.7	428.6	439.8	457.9	474.0
Coefficient of diffusion resistance (-)	4.42	4.10	4.58	4.59	5.23	5.53	5.77	5.69	6.49	7.37

**Table A9:** Detailed results of water vapor permeability tests of F samples.

Measured Parameter (Unit)	F690	F720	F750	F780	F810	F840	F870	F900	F930
Volumetric density at RH 50% (kg/m^3^)	352.3	390.8	393.8	403.3	429.1	441.3	453.4	463.8	507.4
Coefficient of diffusion resistance (-)	5.85	6.14	6.41	6.44	7.59	7.46	7.99	8.71	10.10

### Appendix A.4

**Table A10:** Detailed results of specific heat test of R samples.

Measured Parameter (Unit)	R1	R2	R3	R4	R5	Av.	Std. Dev.
Specific heat at approx. 60 °C (J/(g·K))	1.320	1.237	1.241	1.241	1.259	1.260	0.035
Specific heat at approx. 30 °C (J/(g·K))	0.756	0.810	0.791	0.775	0.732	0.773	0.030
Specific heat at approx. 20 °C ^1^ (J/(g·K))	0.590	0.670	0.667	0.670	0.551	0.629	0.056

^1^ Linear extrapolation.

**Table A11:** Detailed results of specific heat test of W samples.

Measured Parameter (Unit)	W1	W2	W3	W4	W5	Av.	Std. Dev.
Specific heat at approx. 60 °C (J/(g·K))	1.208	1.213	1.180	1.168	1.248	1.204	0.031
Specific heat at approx. 30 °C (J/(g·K))	0.843	0.838	0.817	0.792	0.885	0.835	0.034
Specific heat at approx. 20 °C ^1^ (J/(g·K))	0.699	0.728	0.685	0.682	0.756	0.710	0.031

^1^ Linear extrapolation.

**Table A12:** Detailed results of specific heat test of F samples.

Measured Parameter (Unit)	F1	F2	F3	F4	F5	Av.	Std. Dev.
Specific heat at approx. 60 °C (J/(g·K))	1.185	1.165	1.219	1.178	1.145	1.178	0.027
Specific heat at approx. 30 °C (J/(g·K))	0.878	0.838	0.912	0.858	0.829	0.863	0.033
Specific heat at approx. 20 °C ^1^ (J/(g·K))	0.762	0.701	0.762	0.730	0.689	0.729	0.034

^1^ Linear extrapolation.
